# Beneficial Use of MIBC in Metakaolin-Based Geopolymers to Improve Flowability and Compressive Strength

**DOI:** 10.3390/ma13173663

**Published:** 2020-08-19

**Authors:** Sujeong Lee, Byoungkwan Kim, Joobeom Seo, Shinhu Cho

**Affiliations:** 1 Mineral Resources Research Division, Korea Institute of Geoscience and Mineral Resources, Daejeon 34132, Korea; joobeomi@kigam.re.kr; 2 Department of Resources Recycling Engineering, University of Science & Technology, Daejeon 34113, Korea; Kwan9282@gmail.com; 3 Advanced Materials Research Team, Hyundai Motor Group, Uiwang 16082, Korea; s.cho@hyundai.com

**Keywords:** methyl isobutyl carbinol, flowability, metakaolin-based geopolymers, compressive strength

## Abstract

Superplasticizers (cement concrete water reducers) are applied to improve the flowability of calcium-rich, alkali-activated materials, with inconsistent results. However, superplasticizer applications are limited in metakaolin-based geopolymers. The possibility of using polycarboxylate superplasticizers and methyl isobutyl carbinol (MIBC) to ameliorate the flowability of metakaolin-based geopolymers was investigated. The ratio of metakaolin, fumed silica, NaOH or KOH, and water in geopolymers at a Na_2_O or K_2_O:Al_2_O_3_:SiO_2_:H_2_O ratio = 1:1:4:10 or 1:1:4:11 was maintained in the formulations. In this study, ether- or ester-based polycarboxylate superplasticizers did not improve the workability of fresh metakaolin-based Na-geopolymers. A low MIBC dose (0.5 wt.% of metakaolin) improved the flowability by 19% and additionally increased the 7-day compressive strength by 22% from 68 to 83 MPa for plain Na-geopolymers. The entrained fine froths produced by adding MIBC during mixing likely reduced friction between metakaolin particles, and the slurry became more workable. Hence, the geopolymer mixture with an improved flowability became more homogenous, which ensured more extensive metakaolin dissolution and hydrolysis. A low MIBC dose could be effective for Na-geopolymers with dual benefits of improved workability and enhanced compressive strength.

## 1. Introduction

Geopolymers, consisting of randomly disordered silicon and aluminum tetrahedrons bonded by oxygen atoms, are formed by combining an alkaline solution with reactive aluminosilicate powder, in particular, metakaolin or low-calcium fly ash, at ambient or low temperatures [1,2]. The primary application of geopolymers includes reduced-CO_2_ construction materials as an alternative to Portland-based cements [2], functioning as a monolithic refractory [3] and heat-resistant binder [4]. Geopolymers are typically synthesized from potassium or, more commonly, sodium as the alkali cation and are considered precursors to ceramic manufacturing [5,6]. Metakaolin-based geopolymers reacted with sodium activators have a higher viscosity than those reacted with potassium activators [7]. The high viscosity of sodium silicate solutions prohibits the thorough mixing required for producing a homogeneous slurry and reduces the workability. In terms of workability, potassium hydroxide is preferred over sodium hydroxide, but it is more expensive than sodium hydroxide. Sodium hydroxide is the most commonly used hydroxide activator in geopolymer synthesis, being both the cheapest and most widely available of the alkali hydroxides [2]. Therefore, an admixture is needed to increase the flowability of fresh metakaolin-based geopolymer paste made with sodium hydroxide.

Cement particles are highly likely to agglomerate due to the attractive forces between fine particles. Cement particle flocculation or dispersion can influence the setting behavior, chemical shrinkage and capillary porosity depercolation by substantially decreasing the hydration degree at the initial setting time [8]. Various chemical admixtures have been developed in the cement concrete industry since the 1930s to reduce the water/cement ratio while maintaining a suitable workability and to produce stronger and more durable materials [9]. Superplasticizers are chemical admixtures used for reducing the water demand and are currently essential to cement concrete formulations. Polycarboxylates (PCEs) are comb-shaped superplasticizers and are known to dramatically reduce the water demand in cement concrete [9]. PCEs are steric admixtures, and their dispersing ability is due to steric hindrance effects rather than electrostatic repulsion [10]. Mutual repulsion of the particles is governed by the solubility of the polymeric tails in the liquid [10]. The steric hindrance is expected to affect geopolymers or alkali-activated materials. Geopolymers can have slightly higher pH values than ordinary Portland cement, and the chemical behavior of superplasticizers with various alkali activating solutions seems to be different than that of cement.

Many studies have added superplasticizers to calcium-rich precursors, such as blast furnace slag or high-calcium fly ash [11,12,13,14,15,16,17,18,19,20], but the use of superplasticizers in metakaolin- or low-calcium fly ash-based geopolymers activated by sodium hydroxide or silicates is very limited [21,22,23,24]. In addition, it has not been reported whether plasticizers or superplasticizers applied in geopolymers or alkali-activated materials function as efficiently as when they are applied in cement concrete. For instance, a recent study reported that the addition of a polycarboxylic ether-based superplasticizer improved the flowability of fly ash-based geopolymers without compromising the final strength of the hardened material, which was determined based on a performance assessment of a range of natural and synthetic polymers [10]. The flowability, however, was only slightly improved, and the effect of the additional water present in the superplasticizers was not even considered. The superior dispersing ability of superplasticizers is the key factor for the workability of high-performance and self-consolidating concrete with a water/cement ratio as low as 0.3 or lower [9]. If the flowability is only slightly improved, the use of superplasticizers does not satisfy their original purpose. Superplasticizers can have adverse effects on alkali-activated materials, such as retarding the activation process, failing to improve flowability and even reducing the mechanical strength of alkali-activated slag [15,16,20,25]. On the other hand, certain superplasticizers are not chemically stable, as evidenced by the precipitation of the sulfonated melamine formaldehyde superplasticizer in a KOH solution [26], or the rapid degradation in an alkaline environment [13,27]. In alkali-activated slag systems, the need to develop new chemically stable superplasticizers has emerged to enhance the flowability of slag cement at high pH levels [25].

This paper aims to evaluate the efficiency of the water-reducing performance of polycarboxylate superplasticizers and methyl isobutyl carbinol (MIBC) in metakaolin-based geopolymers activated with sodium hydroxide. The geopolymers investigated here were produced through room-temperature activation of metakaolin with sodium hydroxide and fumed silica. By dissolving fumed silica in an NaOH solution, the optimum formulation of metakaolin-based geopolymers [28] can be achieved. MIBC, which has not been used in metakaolin-based geopolymer systems, and three PCEs were applied during the mixing process. In addition, the compressive strength values of pure Na- and K-geopolymers at different water contents and MIBC-containing Na-geopolymers are compared.

## 2. Material and Methods

### 2.1. Characterization of Metakaolin

Metakaolin MetaMax (BASF, Ludwigshafen, Germany) was adopted to produce geopolymers cured at ambient temperature. X-ray diffraction (XRD) patterns of the representative metakaolin samples were obtained with a D8 Advance diffractometer (Bruker-AXS, Karlsruhe, Germany) over a 2θ range from 5 to 80°. The metakaolin was milled in a micronizing mill (McCrone, Westmont, IL, USA) for 5 min prior to XRD.

The chemical composition of the metakaolin was analyzed via X-ray fluorescence spectroscopy (Shimadzu Sequential XRF-1800, Shimadzu, Kyoto, Japan). Three representative samples were prepared as fused beads, and the average of three measurements of the Al_2_O_3_ and SiO_2_ contents was calculated to formulate geopolymers.

To ensure representative sampling, a sieving riffler manufactured by Quantachrome Instruments (Boynton Beach, FL, USA) was used.

### 2.2. Geopolymer Synthesis and Characterization

The chemical composition of the metakaolin-based geopolymers was proposed as M_2_O:Al_2_O_3_:4SiO_2_:11H_2_O or simply as 1:1:4:11 based on previous transmission electron microscopy (TEM) studies, where M is a charge-balancing cation, such as Na^+^ and K^+^ [28]. The Si to Al ratio in this formula is 2.0, and the Na to Al ratio is 1.0. The 1:1:4:11 formula was primarily adopted in this study for producing geopolymer paste. The formula was modified to 1:1:4:10 to validate the improvement of the geopolymer mixture flowability (Table 1).

Sodium hydroxide (Daejung Chemical, assay 97%, Siheung-si, Korea) or potassium hydroxide (Daejung Chemical, assay 93%, Siheung-si, Korea) was dissolved in distilled water using a magnetic stirrer. After the sodium hydroxide or potassium hydroxide solution was cooled, fumed silica (SiO_2_, CAB-O-SIL EH-5, Cabot Corporation, assay 99.99%, Boston, MA, USA) was added to the NaOH or KOH solution. Metakaolin was mixed with an alkali activator solution at a low speed for 2 min and at a high speed for 5 min with a Kenwood mixer. The chemical additives were added to the mixture before high-speed mixing.

The geopolymer mixture was then poured into 5-cm cube molds and cured at ambient temperature for 7 days after synthesis. The mean compressive strength of the three samples was measured with an MTS 815 rock mechanics test system (460 tons) at a loading rate of 5.5 × 10^−3^ mm/s (MTS Systems Corporation, Eden Prairie, MN, USA).

### 2.3. Characterization of the Chemical Additives

Commercial superplasticizers based on polycarboxylate polymers (PCE 1, PCE 2, and PCE 3) produced in Korea and an aliphatic alcohol, i.e., MIBC (4-methyl-2-pentanol, assay 98%, Sigma-Aldrich, Saint Louis, MO, USA) were tested as water reducers in metakaolin-based geopolymers. PCE 1, PCE 2 and PCE 3 are pale amber-colored liquids, and MIBC is transparent.

The solids content of the superplasticizers was determined by subtracting the amount of evaporated water at 160 °C for 30 min from the total sample weight, i.e., 2 g, as determined with a moisture analyzer (MB 45, OHAUS, Parsippany, NJ, USA). The solids fraction of the superplasticizers was used for Fourier transform infrared spectrometry (FT-IR). Fifteen grams of superplasticizer was immersed in a rotating flask and dried in a water bath designed to maintain the liquid temperature at 80 °C until the water fraction of the superplasticizer was completely evaporated under vacuum. Attenuated total reflectance (ATR)-FT-IR spectra were obtained (Nicolet 6700, Thermo Fisher Scientific, Waltham, MA, USA) over the range of 4000–650 cm^−1^.

### 2.4. Evaluation of the Flowability

**Figure 1:**
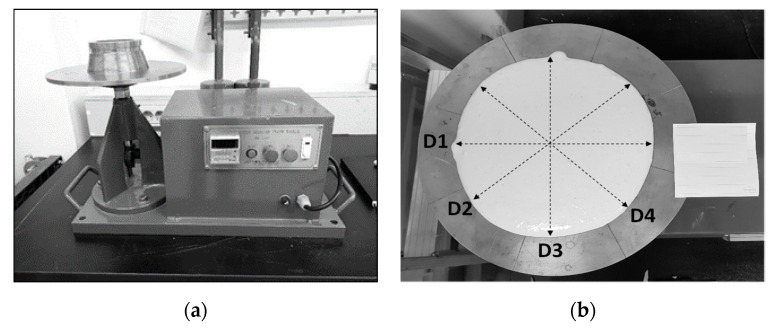
(**a**) Motorized flow table for flowability testing. The conical mold has a 100-mm base diameter, 70-mm top diameter and 50-mm height. (**b**) The average of the four spread diameter values from D1 to D4 is presented as the flow diameter in Table 1.

The flowability of the fresh geopolymer mixtures was evaluated via the flow table test in accordance with KS L 5111. The flow mold was lifted 1 min after geopolymer addition, and the spread diameter was measured after dropping the table 25 times over 15 s (Figure 1a). The average flow diameter was calculated from four spread diameter measurements from D1 to D4 (Figure 1b).

## 3. Results and Discussion

### 3.1. Characteristics of the Metakaolin

**Figure 2:**
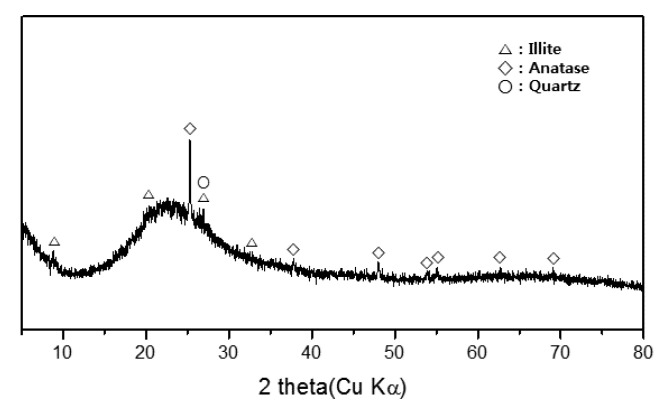
X-ray diffraction pattern of the metakaolin used in this study. It contains crystalline phases such as illite (an aluminosilicate), anatase and quartz (crystalline silica).

XRD revealed that the tested metakaolin contained amorphous and crystalline phases, including quartz, illite and anatase (Figure 2). The major chemical composition of metakaolin was SiO_2_ and Al_2_O_3_ (Table 2). The theoretical composition of anhydrous metakaolin is 54.0% SiO_2_ and 46.0% Al_2_O_3_ based on the theoretical composition of kaolinite, which is composed of 46.54% SiO_2_, 39.50% Al_2_O_3_ and 13.96% H_2_O [29]. The SiO_2_ and Al_2_O_3_ concentrations of 52.6% and 43.8%, respectively, were considered to be fully reactive for formulating geopolymers, even though these values include small amounts of crystalline SiO_2_ and Al_2_O_3_ as quartz and illite, respectively.

### 3.2. Characteristics of the Chemical Additives

**Figure 3:**
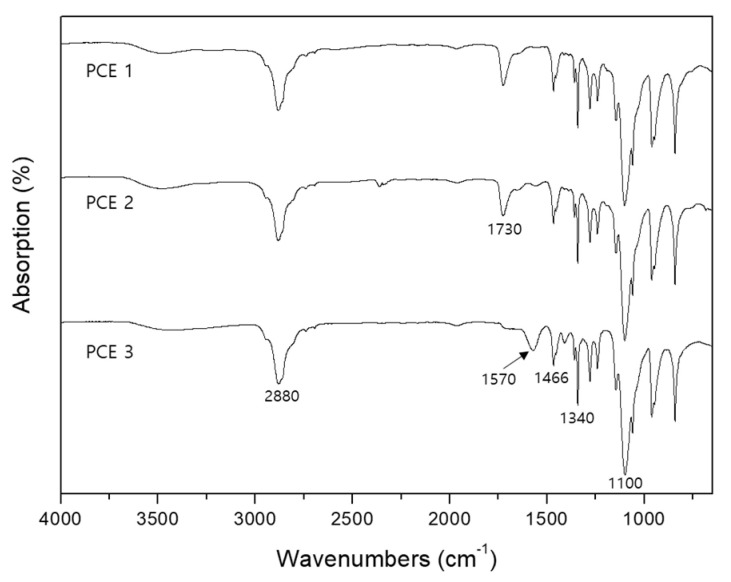
FT-IR spectra of the polycarboxylate superplasticizers used in this study. Superplasticizers PCE 1 and PCE 2 contain ester side chains, while PCE 3 includes ether side chains.

The C=O stretching vibration band at 1730 cm^−1^ in the FT-IR spectra of PCE 1 and PCE 2 indicated that they were ester-based superplasticizers [30] (Figure 3). PCE 3 was estimated to consist of an ether structure because of the asymmetric stretching of C-O-C at 1100 cm^−1^, while the band at approximately 1570 cm^−1^ presumably occurred due to COO^−^ and Na^+^ [31,32]. The bands at 2880, 1466 and 1340 cm^−1^ were assigned to the C-H stretching vibration [32].

The solids content of the PCEs was approximately 16% for PCEs 1 and 2 and approximately 18% for PCE 3. This means that additional water is introduced to the metakaolin-based geopolymer mixture, depending on the PCE dose. The resultant final water content in the geopolymer mixtures was calculated to consider the water content in the superplasticizers (Table 1 and Table 3). The MIBC amount was included in the water content, depending on its dose.

### 3.3. Flowability Enhancement Induced by MIBC

The flowability of the fresh geopolymers deteriorated as the water content decreased. The spread diameter of the 1:1:4:11 Na-geopolymer with a water content of 32.4% was 227 mm and dropped to 159 mm for the 1:1:4:10 Na-geopolymer with a water content of 30.3% (Table 1). The PCE superplasticizers used in this study, PCEs 1, 2 and 3, had little effect on increasing the workability, not only at a metakaolin dose of 5.1 wt.% but also at lower doses (Table 1). When PCE 3 was added at a dosage of 10.2%, the spread diameter increased to 171 mm. However, the additional water present in the PCE solution contributed to the total water content of the mixture (32.3%) and eventually resulted in a better workability (Table 1). When PCE 3 was added at low dosages, namely, 0.3, 0.5 and 1.0%, the spread diameters were not very different from those of the pure geopolymers (Table 1).

Geopolymers attain a far lower yield stress than typical cement paste [33]. Particularly, the cohesion of the calcium-silicate-hydrate (C-S-H) phase is much stronger than the cohesion of the early aluminosilicate gel produced in the geopolymer reaction, and superplasticizers have no effect on geopolymer paste [33]. Metakaolin-based geopolymers can be described as Newtonian fluids, as their viscosity is mainly controlled by the high viscosity of the suspending alkaline silicate solution and not by the contribution of the direct contacts between metakaolin particles [33]. Moreover, it has been verified that sodium ions predominantly induce entropic repulsion, while calcium ions give rise to strong electrostatic coupling in lamellar materials, including hydrated cements and clays [34]. The dispersal ability of superplasticizers is due to electrostatic repulsion or steric hindrance rather than entropic repulsion [11]. Entropic repulsion differs from charge repulsion, realizing particle dispersion and preventing flocculation by steric or entropic effects [35]. Because of the fundamental differences between metakaolin-based Na-geopolymers and ordinary Portland cement, the addition of PCEs can be considered to be ineffective. The results shown in Table 3 also support this assumption.

A low dose (0.5%) of MIBC increased the spread diameter of the geopolymer mixture from 159 to 190 mm (Table 1). A dose that was twice as high produced a slightly smaller spread diameter of 184 mm (Table 1). The spread diameters decreased at high doses, in contrast to what occurred at low doses (Table 1). MIBC is an aliphatic alcohol containing 6 carbon atoms, and has been used to generate bubbles and stabilize froth during froth flotation of minerals in the mining industry [36]. The action of aliphatic alcohols such as MIBC remains elusive despite their many years of industrial use [37]. Although there is no ready explanation for the improvement mechanism, the metakaolin-based geopolymers containing 0.5% MIBC clearly became much more workable than pure geopolymers. One possible reason for the improvement effect of MIBC on metakaolin-based geopolymers is that the fine froths, entrained by adding MIBC during mixing, likely reduced friction between metakaolin particles, and the slurry could further spread. MIBC has limited solubility in water, and consequently, adding MIBC in high doses reduced the flow diameter (Table 1), and the extra amount of MIBC, which is insoluble in water, was separated.

### 3.4. Additional Benefit of MIBC on the Compressive Strength

The mean 7-day compressive strength of three 1:1:4:10 Na-geopolymer specimens was 68 MPa (Table 3). With increasing water contents, the compressive strength of the 1:1:4:11 Na-geopolymer decreased to 59 MPa. A lower water content results in a more compact structure and the development of a higher strength [2,38,39]. When MIBC was added at a dose of 0.5%, the compressive strength of the 1:1:4:10 Na-geopolymer increased by 22% from 68 to 83 MPa for pure geopolymers. Compared with the compressive strength of 1:1:4:10 K-geopolymers, namely, 62 MPa, adding 0.5% MIBC to 1:1:4:10 Na-geopolymers was more beneficial in terms of the increased strength and cost competitiveness.

**Figure 4:**
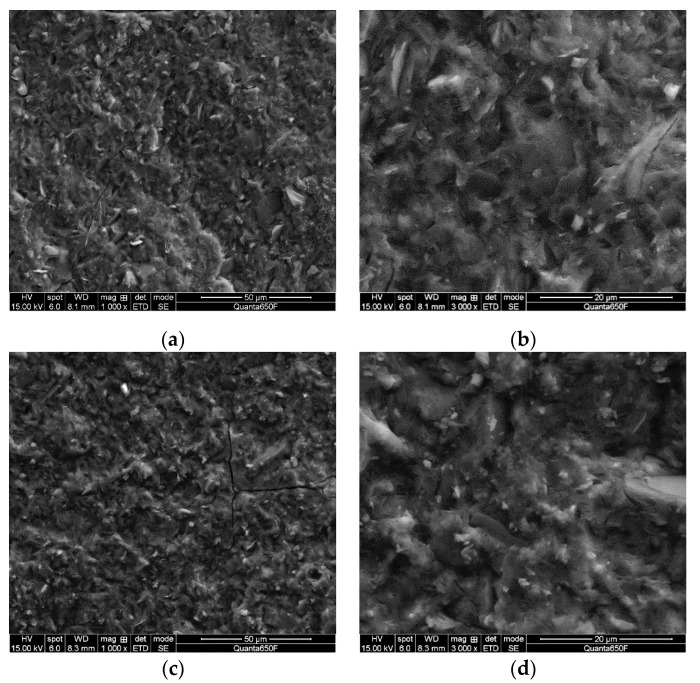
SEM micrographs of fresh fracture surfaces of the Na-geopolymers having a strength of 68 MPa (**a**,**b**) and MIBC-containing Na-geopolymers having a strength of 83 MPa (**c**,**d**). Both specimens exhibit a microstructure comprising dense particulates. No meaningful difference between the microstructures of the fresh fracture surfaces was observed. Cracks could be created during sample preparation or while observing the samples under the high vacuum of SEM.

The microstructure consisting of dense particulates was achieved for both specimens (Figure 4). The addition of MIBC, however, made no perceptible difference in the microstructural evolution of the fresh fracture surfaces of geopolymers, even though the compressive strength increased by 22% (Figure 4). Good mixing is important to obtain homogeneous specimens and to avoid the agglomeration of the mixture for accelerating geopolymerization [40]. The homogeneity of the geopolymer mixtures was crucial to attain a high strength [41]. There is no clear explanation for how MIBC enhanced the compressive strength of geopolymers, but the MIBC-containing geopolymer mixture, with an improved flowability, was likely to be more homogeneous and cohesive than the highly viscous and sticky pure geopolymer mixture. The enhanced homogeneity, caused by the addition of MIBC, ensured the more extensive dissolution and hydrolysis of metakaolin particles. Another possible explanation for the action of MIBC on compressive strength is that less air might be entrapped when the slurry was placed into the molds because of the low viscosity of the slurry. This resulted in decreasing the porosity of the hardened geopolymers, increasing the compactness of the microstructure and, as a result, improving the strength.

As previously mentioned, the geopolymer ingredients were mixed at a high speed for only 5 min in this study, which does not represent thorough mixing. If a high-shear mixer is used with the same formulations, the compressive strength is expected to increase.

## 4. Conclusions

In this study, ether- or ester-based polycarboxylate superplasticizers did not improve the workability of fresh metakaolin-based Na-geopolymers. An aliphatic alcohol, MIBC, improved the flowability of fresh metakaolin-based Na-geopolymer mixtures with an additional strength enhancement effect when 0.5 wt.% metakaolin was added. MIBC did not dramatically reduce the water demand compared to PCEs in cement concrete, but the spread diameter increased by 19% after adding MIBC. The average 7-day compressive strength after synthesis was 68 MPa for the 1:1:4:10 Na-geopolymer. Although the 1:1:4:10 K-geopolymer was much more workable than the 1:1:4:10 Na-geopolymer, the 1:1:4:10 K-geopolymer developed a lower strength of 62 MPa. By adding 0.5% MIBC by weight of metakaolin, the geopolymer mix was less workable than the K-geopolymer, but it developed a much higher strength, i.e., 83 MPa. The entrained fine froths, obtained by adding MIBC during mixing, likely reduced friction between metakaolin particles, and the slurry became more workable. As a result, the geopolymer mix with an improved flowability became more homogenous, which ensured the more extensive dissolution and hydrolysis of metakaolin particles at the early stage of geopolymerization. These results reveal that MIBC, at a low dose, could be effective when added to Na-geopolymers due to the dual benefits of an improved workability and enhanced compressive strength.

## Figures and Tables

**Table 1:** Mixture design of metakaolin-based geopolymers with admixtures. Spread diameter of the fresh geopolymer mixtures was expressed in mm. Methyl isobutyl carbinol (MIBC) gave better performance in terms of flowability, but the higher amount of admixtures resulted in the inverse effect. The slurry of 1:1:4:10 K-geopolymer overflowed from the disc and its flow diameter was assumed to be more than 254 mm.

M_2_O:Al_2_O_3_:SiO_2_:H_2_O (M = Na or K)	Alkaline Activator	Admixture		Water Content (wt.%)	Flow Diameter (mm)
		Type	Dose by Weight of Metakaolin (wt.%)		
**1:1:4:10**	NaOH + fumed silica	-	-	30.3	159 (± 6.24)
		PCE 1 (ester-based)	5.1	31.3	144 (± 4.12)
		PCE 2 (ester-based)	5.1	31.3	136 (± 0.89)
		PCE 3 (ether-based)	0.3	30.3	163 (± 9.39)
			0.5	30.4	163 (± 3.05)
			1.0	30.5	157 (± 7.47)
			5.1	31.3	145 (± 7.81)
			10.2	32.3	171 (± 2.16)
		MIBC	0.5	30.4	190(± 2.83)
			1.0	30.6	184(± 2.46)
			5.1	31.6	179(± 1.05)
			10.2	33.0	175(± 1.30)
**1:1:4:10**	KOH + fumed silica	-	-		>254
**1:1:4:11**	NaOH + fumed silica	-	-	32.4	227 (± 2.60)

**Table 2:** Content of the major elements in the metakaolin used in this study, as determined by X-ray diffraction (XRD).

Oxide	SiO_2_	Al_2_O_3_	Fe_2_O_3_	CaO	MgO	K_2_O	Na_2_O	TiO_2_	MnO	P_2_O_5_	Others
**wt.%**	52.6	43.8	0.30	0.03	0.09	0.16	0.35	1.49	0.01	0.08	0.93

**Table 3:** The water content in each mixture, including the free water in the mixture proportions and the chemical water in the alkali hydroxides, is recalculated to encompass the water content in the polycarboxylates. A low MIBC dose is beneficial to both the flowability and strength development.

Geopolymers	Water Content (wt.%)	Compressive Strength (MPa)
1:1:4:10 Na-GP	30.3	68 ± 4.6
1:1:4:10 Na-GP with MIBC 0.5%	30.4	83 ± 6.0
1:1:4:11 Na-GP	32.4	59 ± 2.5
1:1:4:10 K-GP	32.3	62 ± 4.6
